# CAR T-cell Kinetics, Persistence, and Clinical Outcomes in Adult Patients with Relapsed/Refractory B-cell ALL Treated with Obecabtagene Autoleucel in the FELIX Study

**DOI:** 10.1158/2767-9764.CRC-25-0756

**Published:** 2026-07-15

**Authors:** Claire Roddie, William Day, Meera Raymond, Deborah Cluxton, Jason Caulfield, Heather Dainton-Smith, Brooke McElwee, Yanqing Hu, Karamjeet S. Sandhu, Eleni Tholouli, Aaron C. Logan, Paul Shaughnessy, Deborah Yallop, Pere Barba, Manuel Guerreiro, Jean A. Yared, Katharine Hodby, Bijal D. Shah, Wolfram Brugger, Pierre Lao-Sirieix, Michael R. Bishop, Daniel J. DeAngelo, Elias Jabbour, Jae H. Park

**Affiliations:** 1 University College London Cancer Institute, London, United Kingdom.; 2 Autolus Therapeutics, London, United Kingdom.; 3 Autolus Therapeutics, Rockville, Maryland.; 4 City of Hope National Medical Center, Duarte, California.; 5 Manchester Royal Infirmary, Manchester, United Kingdom.; 6 Hematology, Blood and Marrow Transplantation, and Cellular Therapy Program, University of California San Francisco, San Francisco, California.; 7 Sarah Cannon Transplant and Cellular Therapy Program, Methodist Hospital, San Antonio, Texas.; 8 King’s College Hospital NHS Foundation Trust, London, United Kingdom.; 9 Hospital Universitari Vall d’Hebron-Universitat Autónoma de Barcelona, Barcelona, Spain.; 10 Hospital Universitari i Politècnic La Fe, Valencia, Spain.; 11 University of Maryland, Baltimore, Maryland.; 12 University Hospital Bristol, Bristol, United Kingdom.; 13 Moffitt Cancer Center, Tampa, Florida.; 14 Autolus Therapeutics, Munich, Germany.; 15 The David and Etta Jonas Center for Cellular Therapy, University of Chicago, Chicago, Illinois.; 16 Department of Medical Oncology, Dana-Farber Cancer Institute, Boston, Massachusetts.; 17 University of Texas MD Anderson Cancer Center, Houston, Texas.; 18 Memorial Sloan Kettering Cancer Center, New York, New York.

## Abstract

Assessments of chimeric antigen receptor (CAR) T-cell pharmacokinetics by flow cytometry (FC) and a droplet digital PCR (ddPCR) assay were compared in adult patients with relapsed/refractory (R/R) B-cell acute lymphoblastic leukemia (B-ALL) following treatment with obecabtagene autoleucel (obe-cel) in the phase Ib/II FELIX study (NCT04404660). CAR T-cell persistence and B-cell aplasia (BCA) were then correlated with event-free survival (EFS). Peripheral blood (PB) samples collected from 127 obe-cel–infused patients were tested by FC and ddPCR. The Spearman correlation coefficient was used to measure the correlation between the number of CAR T–positive cells by FC and ddPCR. The impact of CAR T-cell persistence and BCA (B cells <20 cells/μL by FC in PB) on EFS was assessed using Cox proportional hazards regression. ddPCR was observed to statistically correlate with both the surface (0.60, *P* < 0.0001) and intracellular FC assays (0.74, *P* < 0.0001). A higher sensitivity for detecting CAR T–cell positive samples was observed with ddPCR, with 58.8% and 41.3% of samples negative by surface and intracellular FC, respectively, being positive by ddPCR. Loss of CAR T-cell persistence (HR, 2.7; 95% CI, 1.4–5.4) and, to a lesser degree, B-cell recovery (HR, 1.7; 95% CI, 0.7–3.8) as time-dependent variables and at month 3 were associated with poorer EFS. ddPCR demonstrated enhanced sensitivity over FC methods for detection of obe-cel persistence. Additionally, ongoing persistence and BCA were associated with longer EFS and may be taken into consideration, together with clinical parameters, in informing decision making.

**Significance:**

In patients with R/R B-ALL treated with obe-cel, ddPCR assessment of CAR transgene levels produced results consistent with FC while providing superior sensitivity. ddPCR-detected CAR T-cell persistence at month 3 and at any time after infusion correlated with longer EFS versus loss of persistence, therefore potentially informing clinical decision making.

## Introduction

CD19-directed chimeric antigen receptor (CAR) T-cell therapy has emerged as a promising treatment for a range of B-cell hematologic malignancies (1–3), including relapsed/refractory (R/R) B-cell acute lymphoblastic leukemia (B-ALL; refs. 4–10). Obecabtagene autoleucel (obe-cel) is an autologous 4-1BB-ζ CD19-directed CAR T-cell therapy (8, 9), designed using a unique CAT19 binder with substantially lower CD19 binding affinity (>40-fold) compared with the more commonly used FMC63 binder (11, 12). Obe-cel’s fast off-rate results in robust CAR T-cell expansion with reduced infusion-related toxicity and enhanced long-term persistence (8, 9, 11).

The US Food and Drug Association has approved obe-cel in adult patients with R/R B-ALL based on results from the pivotal phase Ib/II FELIX study (NCT04404660; refs. 9, 13). Patients treated with obe-cel had a high rate of overall remission [78%; 95% confidence interval (CI), 70–85], defined by best response of complete remission (CR) or CR with incomplete hematologic recovery (CRi) and a favorable safety profile characterized by a low incidence of grade ≥3 cytokine release syndrome (CRS) and immune effector cell-associated neurotoxicity syndrome (ICANS; ref. 9).

Quantification of the proportion and absolute number of CAR T cells is critical for understanding the expansion and persistence of these cells in patients following CAR T-cell therapy. Alongside measurable residual disease (MRD) eradication (14), CAR T-cell expansion and/or persistence seem to be key factors in treatment success and correlate with clinical outcomes in patients with B-cell malignancies, including in adult patients with R/R B-ALL (3, 5, 9, 10, 15–19). CAR T-cell persistence seems to be of even more importance in CAR designs that incorporate the 4-1BB costimulatory domain compared with those that use the CD28 costimulatory domain (20–22). Furthermore, B-cell recovery/loss of B-cell aplasia (BCA), an indirect marker commonly used as a surrogate for functional CAR T-cell persistence (6), is associated with a higher relapse risk and has shown potential in identifying patients who require consolidative stem cell transplant following CAR T-cell therapy (23). Application of BCA as a prognostic tool is limited by the proliferative capacity of CAR T cells and therefore may be CAR design-specific (24). Additionally, patients with high expansion are also more likely to experience adverse events, including CRS or ICANS (5, 9, 10, 16–19, 25). In light of these considerations, establishing a highly sensitive detection strategy for obe-cel CAR T cells may facilitate the prognostication of long-term outcomes in patients.

Different approaches and bioanalytic assays can be utilized for detecting CAR T cells and monitoring their kinetics (26). Traditionally, multiparametric flow cytometry (FC) has been considered the standard for monitoring CAR T cells (26, 27). As whole cells are analyzed, not only can FC be used to monitor CAR T-cell expansion and persistence but it also enables characterization of the CAR T-cell phenotype by concomitant analysis of other immune protein markers (28). FC is nevertheless prone to variability due to sample quality, operator handling, and reagent choice (29). Additionally, FC data require manual gating to identify the cell population of interest, relying on controls and expert interpretation to set accurate gating limits. Polymerase chain reaction (PCR)-based methods to detect the CAR transgene have proven to be highly sensitive and require a minimal amount of patient sample material. Specifically, real-time PCR (RT-PCR) assays that correlate with FC results have been developed (26); however, RT-PCR assays require the use of external references and standards. More recently, methods for monitoring CAR T-cell expansion and persistence using droplet digital PCR (ddPCR) have been described (17, 28, 30). This method enables absolute quantification without the need for external references or standard curves, such as those required in RT-PCR (28, 31), and has shown higher sensitivity than RT-PCR (31), offering a sensitive, precise, and reproducible method that may expand on the clinical applications of CAR T-cell monitoring (28).

Here, we describe a specific ddPCR assay that was designed for the monitoring of obe-cel kinetics and compare results from this assay with results from surface and intracellular FC assays, using matched patient samples from the FELIX study. Furthermore, we investigate the relationship between obe-cel pharmacokinetics, pharmacodynamics, and clinical outcomes in adult patients with R/R B-ALL. The aim of this analysis is to explore the role that assay sensitivity plays in the longitudinal monitoring of CAR T cells and to better understand pharmacokinetic factors that may influence clinical outcomes in order to support clinical decision making.

## Materials and Methods

### FELIX study and obe-cel treatment

The safety and efficacy of obe-cel in patients aged ≥18 years with CD19^+^ R/R B-ALL was investigated in the phase Ib/II FELIX study (NCT04404660); details of the study, including design, representativeness of the study population, and results, have been published elsewhere (9). Following relevant Institutional Review Board approval, this study was carried out in accordance with the principles founded in the Declaration of Helsinki and Good Clinical Practice guidelines, and written informed consent was obtained from all patients.

### Sample selection and assays

Peripheral blood (PB) samples were collected for surface and intracellular FC assays and/or ddPCR prior to obe-cel infusion on day −6, following obe-cel infusion on days 1, 3, 6, 9, 12, 15, 22, and 28, and months 2, 3, 4, 6, 9, 12, 15, 18, 21, and 24, and then every 6 months until the end of the FELIX trial. Parallel FC and ddPCR were performed on postinfusion samples from 127 patients.

The primary assay used to measure CAR transgene levels [vector copy number (VCN)] in samples from the FELIX trial was a ddPCR assay designed and optimized to provide the highest sensitivity possible for VCN detection in PB after obe-cel infusion. Both the surface and intracellular FC assays were comprehensive 12-color assays primarily designed for assessing CAR T-cell immunophenotype, differentiation state, and exhaustion status rather than CAR T-cell monitoring. The sampling for the two technologies reflects the differences in prioritization in the FELIX trial with an increased number of time points sampled for ddPCR (up to 19 in the 24 months after infusion) compared with FC (up to nine in the 24 months after infusion).

### CAR T-cell enumeration by FC

Immunophenotypic enumeration was assessed using an in-house anti-idiotype antibody to the CD19 CAR construct (CAT19 idiotype; Autolus Therapeutics). A two-part assay was developed to detect both surface and intracellular CARs, which enabled the absolute count of CAT19 CAR T cells/μL in blood.

For analysis using the surface FC assay, 100 μL of patient PB was transferred to a BD Trucount tube for direct immunofluorescence staining using a lyse/no-wash protocol (BD Biosciences). Cells were blocked with human Fc receptor blocking reagent (Miltenyi Biotec) and incubated for 10 minutes at room temperature. An antibody mastermix (Supplementary Table S1) was added at a volume of 55 μL to the PB. The blood was incubated at 4°C in the dark for 20 minutes. The live/dead staining and lysis steps were combined by adding Fixable Viability Stain 700 (BD Biosciences) at a concentration of 1 in 2,000 to the lysis buffer (BD Pharm Lyse), made by diluting the stock reagent 1 in 10 with ultrapure water. The combined buffer was added to the PB to a final volume of 1 mL and incubated in the dark at room temperature for 15 minutes. Along with the fully stained patient sample, a fluorescence minus one control for the CAR T-cell detection idiotype was generated along with CAR-positive and -negative control cell lines.

To assess CAR T cells using the intracellular FC assay, 5 mL of patient PB was added to lysis buffer (BD Pharm Lyse, BD Biosciences). The patient sample was then washed and resuspended in 100 μL of live/dead staining solution (Fixable Viability Stain 700; diluted 1:1,000 μL in PBS) for 10 minutes at room temperature in the dark. The live/dead staining solution was washed off using staining buffer, and the cells were blocked with human Fc Receptor Blocking Reagent (Miltenyi Biotec), resuspended in surface antibody master mix (Supplementary Table S2), and then incubated in the dark at 4°C for 20 minutes. After incubation, the samples were washed with staining buffer and fixed in 100 μL of fixation solution (Thermo Fisher Scientific) for 20 minutes at 4°C in the dark. Following incubation with the fixation solution, 1 mL of permeabilization solution (Thermo Fisher Scientific) was added to the sample which was then incubated for 5 minutes. The sample was then washed, resuspended in intracellular master mix (Supplementary Table S3), and incubated for 30 minutes at 4°C in the dark. Excess antibody was then removed, and the sample was resuspended in a final volume of 350 μL for acquisition on the BD Lyric flow cytometer (BD Biosciences). The fully stained patient sample, four fluorescence minus multiple controls for setting gating limits (Supplementary Table S4), along with CAR-positive and CAR-negative control cell lines, were run for each patient time point.

To determine the number of CAT19 CAR T cells/μL for the surface assay, the number of BD Trucount beads (BD Biosciences) in the pellet was divided by the number of beads acquired. This ratio was multiplied by the number of events in the CD3^+^/CAT19+ gate and then divided by the total volume of blood used in the assay (100 μL). For the intracellular assay, PB cells were stained intracellularly with CAT19 idiotype and the proportion of CD3^+^/CAT19+ cells determined. The number of CAT19 CAR Tcells/μL was then calculated using the absolute count of CD3^+^ cells/μL from the surface assay divided by the surface assay blood volume (100 μL). Cells were counted using the Becton Dickinson (BD) FACSLyric FC system incorporating the BD cytometer and tracking beads for automated cytometer setup. Data were analyzed using the BD FACSuite software. The lower limit of quantitation (LLoQ) for both the surface and intracellular FC assays was assessed by spiking known quantities of CAR T cells into PB; quantifiable measurements (above the LLoQ) defined CAR T-cell positivity, and measurements not quantifiable defined CAR T-cell negativity (below the LLoQ). Samples were defined as being outside the window of stability if they were >72 hours after collection.

### CAR T-cell enumeration by ddPCR

VCN per diploid genome was determined through duplex detection of the lentiviral vector Ψ (L-Psi) region and the endogenous reference gene, *RPP30*, using a ddPCR assay developed in a central laboratory. L-Psi was detected in the fluorescein channel, and RPP30 in the hexachlorofluorescein channel. Primers and probes were designed to target the L-Psi sequence and *RPP30* gene (Supplementary Table S5).

Genomic DNA (gDNA) was extracted from PB collected in PAXgene tubes (BD Biosciences) using the QIAamp DSP DNA Blood Mini or Midi Kit (Qiagen), concentrated using an Amicon Ultra 0.5 filter (MilliporeSigma). The extracted DNA was quantified using a Qubit 2.0 fluorometer, and 260/280 ratios measured using a NanoDrop 2000 UV-Vis spectrophotometer (Thermo Fisher Scientific), per the manufacturers’ instructions. ddPCR was performed as a single reaction, using reagents detailed in Supplementary Table S5, to a total volume of 20 μL.

Droplets were generated using the QX200 Droplet Generator (Bio-Rad), according to manufacturer’s instructions, to a final reaction volume of 40 μL. DNA amplification was performed on a C100 Touch Thermal Cycler (Bio-Rad) with the following cycling protocol: denaturation at 95°C for 10 minutes, amplification at 94°C for 30 seconds and 59°C for 1 minute (40 cycles), and an extension step at 98°C for 10 minutes before an indefinite hold at 12°C. The ramp rate was set to 2°C/second before being reduced to 1°C/second at the indefinite hold. The lid was heated to 105°C.

Droplet fluorescence was measured using the QX200 Droplet Reader, and data analyzed using QX Manager v1.2 (Bio-Rad). The thresholds for droplet sensitivity were based on no-template and positive controls. VCN per μg of gDNA was subsequently derived from the ratio of L-Psi and *RPP30* copy numbers. The lower limit of detection (LLoD) and LLoQ were determined using a 15-point dilution series of a synthetic L-Psi template in a constant background of gDNA. Samples were identified as CAR T cell–positive or persistence ongoing if the L-Psi number of copies per reaction was above or equal to the LLoD. PB samples in PAXgene tubes were defined as being outside the window of stability when they were kept at ambient temperature for >14 days or at −20°C for >10 weeks. All reactions were run in technical duplicates.

### BCA

BCA was measured in patients’ PB via FC using BD Multitest 6-color TBNK Trucount Kit (BD Biosciences). BCA (CD45^+^ CD3^−^ CD19^+^ cells) was defined as absolute numbers of B cells <20 cells/μL. Time to B-cell recovery was defined as the days between the first obe-cel infusion and the first time at which ≥20 cells/μL of B cells was measured in PB.

### Statistical analysis

FC and ddPCR were compared using matched samples, defined as samples with both FC and ddPCR data available. Concordance between surface FC and ddPCR, and intracellular FC and ddPCR were assessed using the following parameters: overall percent agreement (%): (number of samples in which ddPCR and FC results were both positive or both negative divided by total number of matched samples) × 100; positive percent agreement (%): (number of samples positive by both ddPCR and FC divided by number of samples positive by FC among the matched samples) × 100; and negative percent agreement (%): (number of samples negative by both ddPCR and FC divided by number of samples negative by FC among the matched samples) × 100. The Spearman correlation coefficient was used to measure the correlation between the number of CAR T–positive cells by FC and VCN by ddPCR.

Landmark analyses of event-free survival (EFS) by CAR T-cell persistence, as measured by ddPCR and intracellular FC, at months 3 and 6 and by BCA, as measured by FC, at month 6 after obe-cel infusion were performed among patients with ongoing remission at those months. Cox proportional hazards regression of EFS was used to estimate hazard ratios (HR) and 95% CIs to evaluate the impact of the loss of CAR T-cell persistence and B-cell recovery on EFS. In this regression analysis, the single, time-dependent explanatory variable was the CAR T-cell persistence status (loss vs. ongoing) or the B-cell status (recovery vs. aplasia). All statistical analyses were descriptive in nature for these *post hoc* secondary analyses of the study, and therefore *P* values were not generated.

## Results

### Sample collection

**Figure 1.**
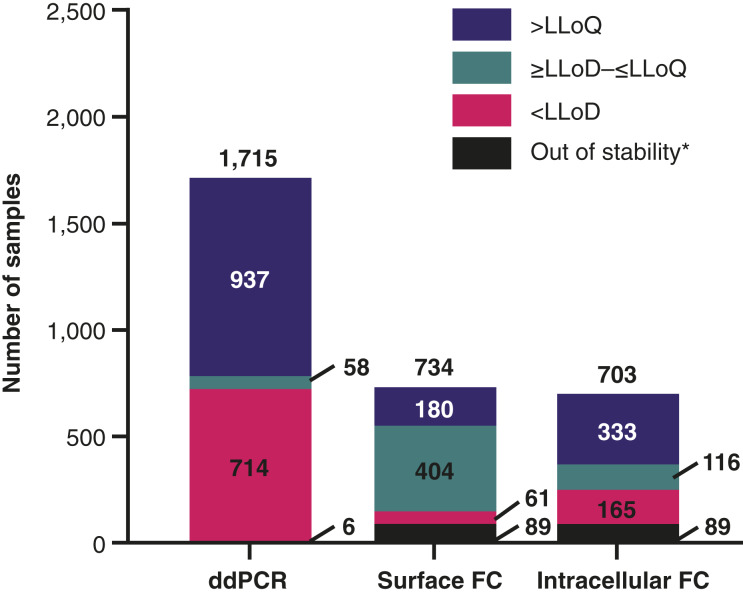
Breakdown of all samples collected for each CAR T-cell enumeration method, including the number of samples that were out of stability*, <LLoD, ≥LLoD to ≤LLoQ, and >LLoQ. *Out of stability is defined as blood samples >72 hours after draw for FC and PB samples in PAXgene tubes kept at ambient temperature for >14 days or at −20°C for >10 weeks for ddPCR.

In the FELIX trial, 127 patients were infused with obe-cel. Baseline characteristics for the 127 patients were reported elsewhere (9); the median age was 47 years (range, 20–81), 52% were male, and 29.9% were Hispanic or Latino. After a median follow-up of 21.5 months (range, 8.6–41.4), a total of 1,715 samples were collected from obe-cel–infused patients at all time points for ddPCR CAR T-cell enumeration, 734 for surface FC, and 703 for intracellular FC (Fig. 1). For ddPCR, 937 of 1,715 samples (54.6%) were above the LLoQ, 58 of 1,715 (3.4%) had detectable CAR-positive T cells measuring above the LLoD but below the LLoQ, and 714 of 1,715 (41.6%) measured below the LLoD, with only six samples (0.3%) outside the stability window and therefore unable to be measured. Of the 734 samples for surface FC, 180 (24.5%) were above the LLoQ, 404 (55%) were above the LLoD but below the LLoQ, 61 (8.3%) were below the LLoD, and 89 (12.1%) were outside the stability window. For intracellular FC, 333 of 703 samples (47.4%) were above the LLoQ, 116 of 703 (16.5%) were above the LLoD but below the LLoQ, 165 of 703 (23.5%) were below the LLoQ, and 89 of 703 samples (12.7%) were outside the stability window (Fig. 1). A proportion of background signal events falling above the level of LLoD in both surface and intracellular FC assays resulted in false-positive events; therefore, for the FC assays, the LLoQ was used as the metric to perform a comparison with ddPCR. Overall, 590 samples collected from 127 patients who received at least one infusion of obe-cel had matched FC and ddPCR results.

All preinfusion samples were CAR T cell–negative by ddPCR assessment. Samples used for ddPCR were taken from 19 time points, and those for FC were taken from nine time points, over a 24-month period. The calculated LLoQ for the surface and intracellular FC assays were 22.8 cells/μL and 3.1 cells/μL, respectively. The LLoD for the L-Psi assay in duplex with the RPP30 assay was determined to be 11 copies, and the LLoQ was 21 copies (Supplementary Table S5). Of the 590 postinfusion matched samples for FC and ddPCR, CAR-positive T cells were quantifiable by surface FC in 165 (28%) and by intracellular FC in 319 (54.1%) and detectable by ddPCR in 413 (70%) samples (Table 1).

**Table 1.** Analysis of concordance between surface FC or intracellular FC data and ddPCR data in matched samples.

ddPCR vs. surface FC, *n*	Surface FC		
	Positive	Negative	Total
ddPCR	​	​	​
Positive	163	250	413
Negative	2	175	177
Total	165	425	590

ddPCR vs. intracellular FC, *n*	Intracellular FC		
	Positive	Negative	Total
ddPCR	​	​	​
Positive	301	112	413
Negative	18	159	177
Total	319	271	590

Concordance, %	Positive percent agreement	Negative percent agreement	Overall percent agreement
ddPCR vs. surface FC	98.8	41.2	57.3
ddPCR vs. intracellular FC	94.4	58.7	78

CAR T-cell positivity was defined by the LLoQ for the FC assays and LLoD for the ddPCR assay. Positive percent agreement: (number of samples positive by both ddPCR and FC divided by number of samples positive by FC among the matched samples) × 100. Negative percent agreement: (number of samples negative by both ddPCR and FC divided by number of samples negative by FC among the matched samples) × 100. Overall percent agreement: (number of samples in which ddPCR and FC results were both positive or both negative divided by total number of matched samples) × 100.

### Concordance between FC and ddPCR data

Due to the high LLoQ for the surface FC assay, fewer samples were quantifiable compared with ddPCR, leading to a high number of samples that were positive by ddPCR but not quantifiable by surface FC. The surface FC assay had a low overall percent agreement (338/590, 57.3%) and negative percent agreement (175/425, 41.2%) with ddPCR (Table 1). The intracellular FC assay had a higher overall percent agreement with ddPCR (460/590, 78%) compared with surface FC. The primary factor for the difference in overall percent agreement with ddPCR between the two FC assays is the negative percent agreement. The negative percent agreement between intracellular FC and ddPCR of 58.7% (159/271) was higher than that of surface FC compared with ddPCR (175/425, 41.2%). This is attributed to the lower LLoQ measured in the intracellular assay, allowing for improved detection of CAR T cell–positive samples and a closer alignment with negative results from ddPCR.

When a sample was positive by surface and/or intracellular FC, it also tended to be positive by ddPCR (positive percent agreement >90%; Table 1). A higher sensitivity for detecting CAR T cell–positive samples was observed with ddPCR versus FC, with 250 of 425 (58.8%) samples that tested negative by surface FC and 112 of 271 (41.3%) samples that tested negative by intracellular FC being positive by ddPCR. Given the higher percentage agreement between intracellular FC and ddPCR compared with surface FC, together with the greater sensitivity of ddPCR relative to FC, the ddPCR and intracellular FC assays were used to evaluate the association between CAR T-cell persistence and clinical outcomes.

**Figure 2.**
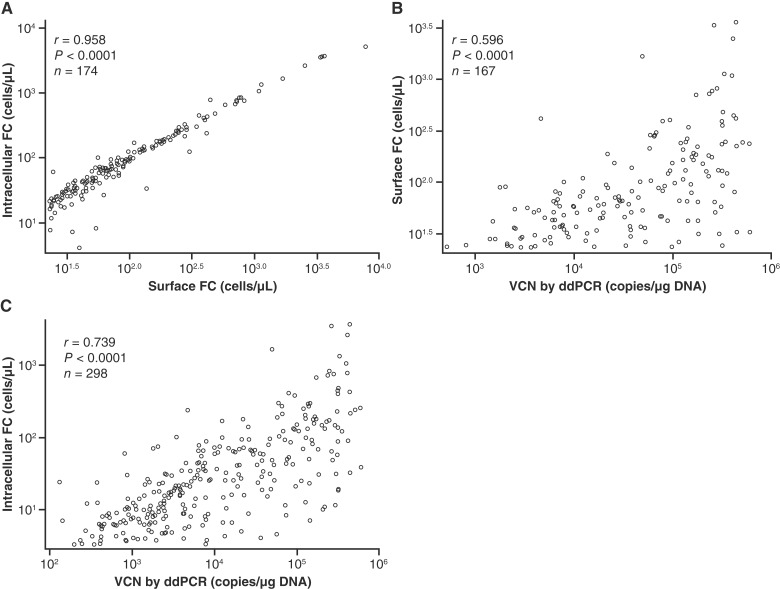
Correlation between (**A**) surface and intracellular FC, (**B**) surface FC and VCN by ddPCR, and (**C**) intracellular FC and VCN by ddPCR. Spearman correlation coefficients reported for all data above the LLoQ.

When using data above the LLoQ, the surface and intracellular FC assays demonstrated a very strong correlation (Spearman correlation coefficient of 0.96, *P* < 0.0001; Fig. 2A). Surface FC demonstrated only a moderate correlation with VCN by ddPCR (Spearman correlation coefficient of 0.60, *P* < 0.0001; Fig. 2B), whereas intracellular FC demonstrated a strong correlation with VCN by ddPCR (Spearman correlation coefficient of 0.74, *P* < 0.0001; Fig. 2C).

### CAR T-cell persistence, BCA, and survival outcomes

**Figure 3.**
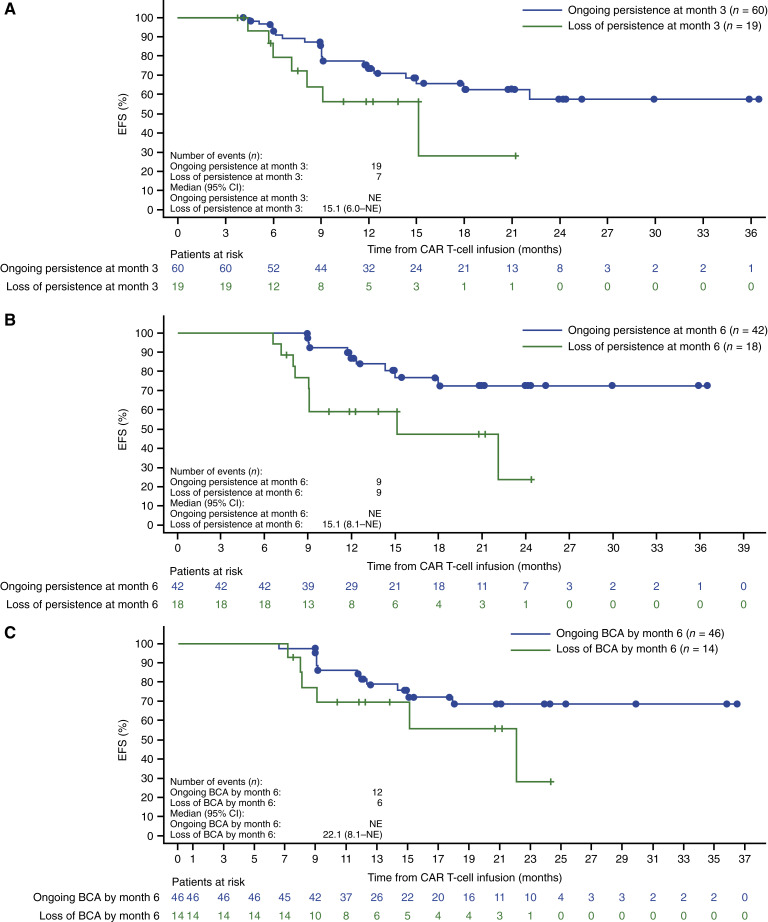
Kaplan–Meier landmark analysis of EFS among patients with ongoing remission without new anticancer therapies by (**A**) CAR T-cell persistence status, by ddPCR, at month 3 (**B**) CAR T-cell persistence status, by ddPCR, at month 6, and (**C**) BCA status at month 6 after obe-cel infusion.

A total of 79 patients at month 3 and 60 patients at month 6 were in ongoing remission (CR/CRi). Continued CAR T-cell persistence was observed in 60 of 79 (75.9%) patients in ongoing remission at month 3 and in 42 of 60 (70%) patients in ongoing remission at month 6. In landmark analyses, ongoing CAR T-cell persistence at month 3 (Fig. 3A) and month 6 (Fig. 3B), measured by ddPCR, was associated with longer EFS compared with patients who had loss of CAR T-cell persistence. The median EFS (95% CI) was 15.1 months [6–not estimable (NE)] and 15.1 months (8.1–NE) in patients who lost persistence by months 3 and 6, respectively, whereas it was not yet reached in patients with ongoing persistence at both time points. The 9- and 12-month EFS probability estimates (95% CI) were 85.4% (72.9–92.4) and 73.5% (59.3–83.4) in patients with ongoing persistence at month 3, whereas in patients who had lost persistence by month 3, EFS probability estimates were 64.2% (33.9–83.4) and 56.2% (26.9–77.6), respectively. The 12-month EFS probability estimate was 87.3% (72–94.5), as described in Roddie and colleagues (9), and the 15-month EFS probability estimate was 77% (58.7–88) in patients with ongoing persistence at month 6 versus 59.3% (33–78.1) at both time points in patients without ongoing persistence at month 6. Landmark analyses of EFS by CAR T-cell persistence, measured by intracellular FC, at months 3 and 6, are presented in Supplementary Fig. S1.

Continued BCA (<20 cells/μL) was observed in 46 of 60 (76.7%) patients in ongoing remission at month 6. In a landmark analysis, longer EFS was observed in patients with ongoing BCA at month 6 (Fig. 3C) compared with patients who had B-cell recovery. The median EFS (95% CI) was 22.1 months (8.1–NE) in patients with B-cell recovery at month 6, whereas it was not yet reached in patients with ongoing BCA. The 12- and 15-month EFS probability estimates (95% CI) were 81.7% (66.6–90.4) and 72.4% (55.3–83.9) in patients with BCA at month 6 versus 69.6% (37.8–87.4) for both time points in patients with B-cell recovery.

**Figure 4.**
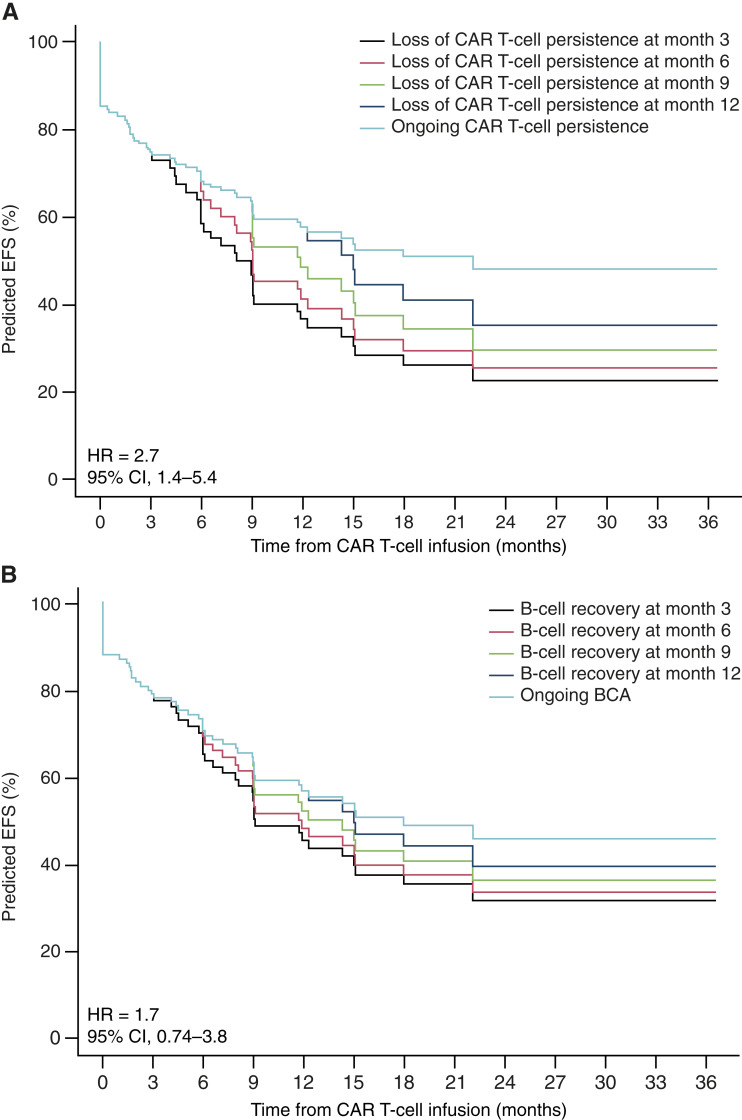
Cox regression analysis showing effect of (**A**) loss of CAR T-cell persistence (by ddPCR quantification of CAR transgene) and (**B**) B-cell recovery (by FC) on EFS (predicted EFS for all infused patients).

**Figure 5.**
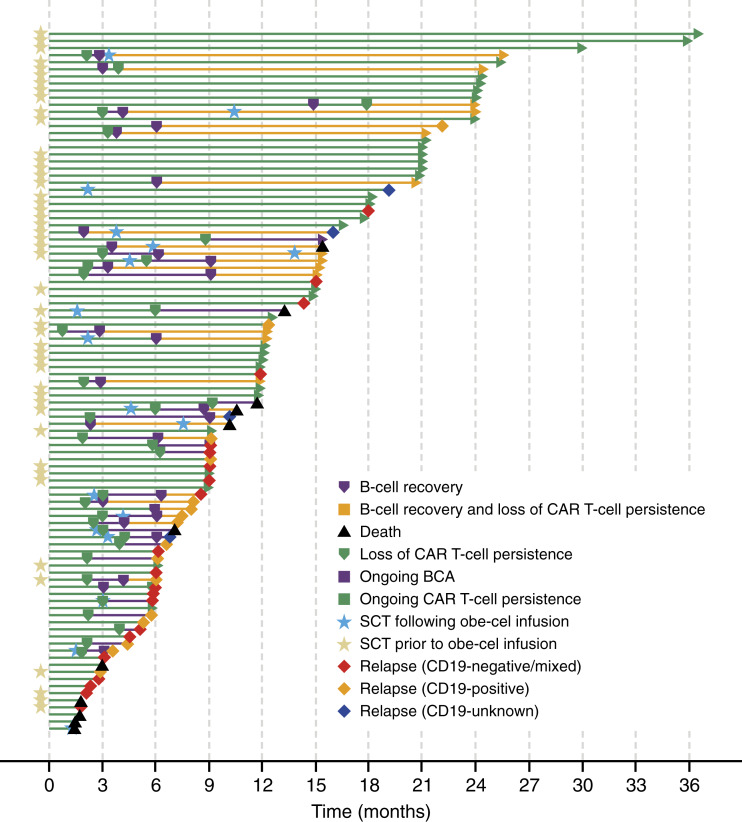
Outcomes following treatment with obe-cel in responding patients, with disease assessment by independent response review committee.

A Cox proportional hazards model showed that loss of CAR T-cell persistence, based on all time points, was associated with a 2.7-fold increased risk of relapse or death compared with ongoing persistence (HR, 2.7; 95% CI, 1.4–5.4; Fig. 4A). Although not significant, B-cell recovery was also associated with an increased risk, with a 1.7-fold increase in relapse or death compared with ongoing BCA (HR, 1.7; 95% CI, 0.7–3.8; Fig. 4B). Overall, a high incidence of both CAR T-cell persistence and BCA was observed in patients with long-term remission (Fig. 5). Similar analyses were performed using BCA thresholds of <10 cells/μL, in line with recently published international consensus guidelines (32), and <50 cells/μL. The results obtained with the <10 cells/μL threshold were consistent with those observed for the <20 cells/μL threshold. However, Cox regression analyses demonstrated a reduced separation between the five predicted EFS curves for ongoing BCA versus B-cell recovery at months 3, 6, 9, and 12, when BCA was defined as <50 cells/μL.

Thirty-two of 40 (80%) patients in remission without non–protocol-specified anticancer therapies had ongoing CAR T-cell persistence (by ddPCR), whereas 33 (82.5%) patients had ongoing BCA (by FC). Time between CAR T-cell persistence loss and B-cell recovery was variable across the patient data set; however, loss of CAR T-cell persistence usually preceded B-cell recovery as the targeted depletion of CD19 is maintained by the presence of the CAR T cells (Fig. 5). Indeed, at month 3, 12 of 19 (63.2%) patients with loss of CAR T-cell persistence had ongoing BCA, whereas this reduced to 4 of 18 (22.2%) at month 6. Most patients with ongoing CAR T-cell persistence had ongoing BCA [58/60 (96.7%) at month 3 and 42/42 (100%) at month 6; Supplementary Table S6].

## Discussion

In this analysis, we compared results of the ddPCR and surface and intracellular FC assays used in the FELIX study for detecting and monitoring obe-cel kinetics in adults with R/R B-ALL. Whereas FC and ddPCR performed similarly in detecting CAR T cell–positive samples, ddPCR demonstrated enhanced sensitivity over both FC methods. We also showed that patients with extended persistence and continued BCA had longer EFS compared with patients who lost persistence or had B-cell recovery, respectively. These data may provide important information to clinicians and potentially affect future clinical decisions.

Multiple groups have utilized ddPCR for the quantification of CAR T cells in lymphoma clinical studies (17, 19, 30), whereas in R/R B-ALL, CAR T-cell expansion and persistence following CAR T-cell therapy have largely been measured using quantitative/qualitative PCR or FC (4, 10, 27, 33). It is important to note that FC allows for additional specificity in the selection of live cells with lymphocyte marker expression (34) and for the collection of information on the cell state by combining markers of activation, differentiation, and exhaustion into a fluorophore panel (35). Furthermore, FC may enable tracking of safety aspects of CAR T-cell treatment, including CRS/ICANS and prolonged cytopenia at critical time points (36). Although ddPCR only enables assessment of CAR T-cell expansion and persistence, an advantage of this assay is that it does not require additional analytic methodology to provide a data readout; this is in contrast to FC, in which manual gating assessment is made for each sample, requiring controls and expert input to produce accurate data.

As multiparametric FC has traditionally been considered the standard method for monitoring CAR T-cell persistence (27); comparison of the ddPCR method with the FC assays ensured robustness of the results. The ddPCR assay designed for FELIX demonstrated increased sensitivity compared with the FC assays used to detect CAR T cells in patients treated with obe-cel. When comparing all data above the LLoQ, a strong correlation between surface and intracellular FC, as well as between intracellular FC and ddPCR, was observed. However, only a moderate correlation was observed between surface FC and ddPCR. A general concordance between methods is supported by the literature, as other studies have demonstrated good agreement between surface and intracellular FC and ddPCR in the detection of CAR T cells with Spearman’s correlation ranging from 0.58 to 1 (30, 37, 38) and Pearson’s correlation of 0.93 (17).

In this investigation, the concordance analysis indicated high positive percent agreement between both FC methods compared with ddPCR (>90%). The relatively low negative percent agreement observed was likely due to the increased sensitivity of the ddPCR assay, leading to categorization of FC-negative samples (below the LLoQ) as “detectable” by ddPCR. Overall, the intracellular FC assay was more sensitive than the surface FC assay for CAR T-cell detection, potentially due to binding interference from the erythrocyte lyse/no-wash protocol used in the surface FC assay. The moderate correlation observed between surface FC and ddPCR was most likely due to the reduced number of matched pairs available, caused by the high LLoQ for surface FC, or due to compounded variation from VCN measurement at higher frequencies as a result of the VCN of lentiviral integrations per cell (39). Also, given the variation in the number of CAR transgene insertion sites within a T-cell population (39), VCN measures will have greater variation compared with an absolute measure such as cells/μL attained using FC. A dedicated FC assay for assessment of CAR T-cell expansion and persistence would likely need further development to be suitable for monitoring CAR T-cell levels.

Whereas ddPCR assays have potential for detecting nonexpressing integrants and persistent CAR T cells without functional activity (40), the continued functional persistence observed following obe-cel infusion would indicate that nonexpressing integrants are not a major contributing factor in this analysis. Of the 40 patients in remission without other non–protocol-specified anticancer therapies, 32 patients had ongoing persistence and 33 patients had ongoing BCA. This suggests that the persisting CAR T cells possess the functional capacity to target CD19-expressing cell populations.

Detection and subsequent monitoring of CAR T-cell kinetics have enabled exploration of the role of CAR T-cell expansion and persistence on outcomes in patients with R/R B-ALL. In pediatric and young adult patients with R/R B-ALL in the ELIANA study, Maude and colleagues (4) found that persistence of tisagenlecleucel in PB was associated with durable clinical responses, with CAR-T persistence observed up to 20 months after infusion (median duration: 168 days). Similarly, in the CARPALL study investigating obe-cel in pediatric patients with R/R B-ALL, after a median follow-up of 14 months, 5/14 patients were in ongoing remission, all of whom had ongoing CAR T-cell persistence (11). In adults with R/R B-ALL, obe-cel persistence was first reported in the phase I ALLCAR19 study, in which 75% (*n* = 3/4) of patients with >2 years of follow-up had ongoing CAR T-cell persistence, assessed using quantitative PCR and FC (8). Additionally, the median duration of obe-cel persistence by ddPCR in patients who achieved remission in the FELIX study (*n* = 99) was 17.8 months (95% CI, 6.2–NE); relapse with ongoing persistence was associated with CD19-negative blasts (9).

Monitoring of CAR T-cell persistence has commonly been performed using BCA as a surrogate, with B-cell recovery associated with increased relapse risk following CAR T-cell therapy (6, 41, 42); however, the duration of CAR T-cell persistence and BCA needed to abate the risk of relapse is unclear (43). In this analysis, the majority of patients who were in remission without non–protocol-specified anticancer therapies at their last follow-up following obe-cel treatment had ongoing CAR T-cell persistence (80%) and BCA (82.5%). Data from this analysis show that ongoing persistence of obe-cel, measured using ddPCR or intracellular FC, at months 3 and 6, or ongoing BCA at month 6, was associated with prolonged EFS compared with loss of persistence or B-cell recovery, respectively. Persistence status as determined by ddPCR showed the greatest difference in median EFS between patients with ongoing versus loss of persistence.

Whereas the median duration of CAR T-cell persistence was 17.8 months, the median duration of BCA was not yet reached in patients who achieved remission (9), suggesting that loss of persistence may be an earlier event than B-cell recovery, and therefore potentially also an earlier indicator of clinical outcomes after obe-cel infusion. In this analysis, 63.2% of patients who lost CAR T-cell persistence by month 3 had ongoing BCA, suggesting that some patients who lost persistence did not have sufficient time to recover from BCA. However, loss of CAR T-cell persistence at month 6 was accompanied by B-cell recovery in most patients. Landmark and Cox proportional hazard regression analyses of EFS by BCA status, using a stricter BCA definition (B cells <10 cells/μL) in line with published international consensus guidelines (32), yielded results consistent with the <20 cells/μL threshold and did not improve alignment with CAR T-cell persistence by ddPCR. However, Cox regression analyses indicated reduced predictive ability of B-cell recovery for risk of relapse or death when BCA was defined as <50 cells/μL.

The results of this analysis, taken together with results from the CARPALL and ALLCAR19 studies, show the potential of obe-cel persistence in predicting clinical outcomes. Data from the ELIANA study have also shown a correlation between persistence of tisagenlecleucel and durable clinical responses (4). In contrast, data from the ZUMA-3 study showed that CAR T-cell persistence following treatment with brexucabtagene autoleucel was lost by month 6 in 79% (*n* = 22/28) of evaluable patients; notably, in ZUMA-3, achieving response and MRD negativity seemed to be associated with CAR T-cell expansion instead (10). Whereas brexucabtagene autoleucel uses the CD28 costimulatory domain (44), both obe-cel (8, 9, 11) and tisagenlecleucel (4) use the 4-1BB costimulatory domain. Taken together, these data suggest that the link between CAR T-cell persistence and better clinical outcomes may be CAR-specific and that it may be related to the design of the CAR and the choice of costimulatory domains (20–22).

Overall, these findings support the potential of this ddPCR assay to identify specific subgroups who may respond differently to treatment with obe-cel through monitoring of CAR T-cell persistence. FC assays developed specifically for CAR T-cell monitoring may also be sufficiently sensitive to be of clinical relevance. Standardized CAR-marking assays, using either FC or PCR methodologies, will be required for assessment of commercially treated patients to enable the potential benefit of including CAR T-cell persistence testing in routine clinical practice. A larger validation cohort using an appropriately developed CAR T-cell monitoring FC assay versus a ddPCR assay would be required to confirm the results shown here. Additionally, a comparison of the impact of persistence monitoring with MRD monitoring on clinical outcomes using real-world evidence would enable further understanding of the role of persistence in predicting clinical outcomes.

Our data show durable CAR T-cell persistence in patients treated with obe-cel and suggest that ongoing CAR T-cell persistence, as measured by ddPCR, and BCA following treatment with obe-cel, are associated with longer EFS. The ddPCR assay designed to monitor obe-cel kinetics demonstrated a higher level of detection of CAR T cell–positive samples, which may be meaningful in the clinical context, considering the correlation of obe-cel persistence with EFS. The status of CAR T-cell persistence in patients treated with obe-cel, in conjunction with other molecular and clinical parameters such as BCA and MRD status, may help inform clinical decision making following obe-cel infusion and help predict treatment success.

## Supplementary Material

Supplementary Table S1 Surface flow cytometry antibody master mix

Supplementary Table S2 Intracellular flow cytometry surface antibody master mix

Supplementary Table S3 Intracellular flow cytometry master mix

Supplementary Table S4 Fluorescence minus multiple controls for setting gating limits

Supplementary Table S5 Summary of L-Psi duplex ddPCR assay

Supplementary Table S6 Summary of CAR T-cell persistence and BCA status at Month 3 and 6

Supplementary Figure S1 Kaplan-Meier landmark analysis of EFS among patients with ongoing remission without new anti-cancer therapies, by CAR T-cell persistence by intracellular flow cytometry at A) Month 3 and B) Month 6.

## Data Availability

The data that support the findings of this study will be made available to qualified researchers for agreed prespecified purposes upon written request after the approval of obecabtagene autoleucel by EU regulatory authorities. All data access requests should be sent to clinicaltrials@autolus.com.
