# Supplementary material for: CAR T-cell Kinetics, Persistence, and Clinical Outcomes in Adult Patients with Relapsed/Refractory B-cell ALL Treated with Obecabtagene Autoleucel in the FELIX Study
Source: Cancer Res Commun. 2026 Jul 15;6(7):1681–92. doi: 10.1158/2767-9764.CRC-25-0756 (PMC13370329; doi:10.1158/2767-9764.CRC-25-0756)
Supplement: Supplementary Table S1 — Surface flow cytometry antibody master mix [file crc-25-0756_supplementary_table_s1_suppst1.pdf]

Supplementary Table S1. Surface flow cytometry antibody master mix.

Antigen	Fluorophore	Clone	Company, catalog and RRID
Brilliant stain buffer plus	-	-	BD Biosciences, Cat# 566385, RRID:AB_2869761
CD19	Brilliant Violet 421	HIB19	BD Biosciences, Cat# 562440, RRID:AB_11153299
CD8	Brilliant Violet 480	RPA-T8	BD Biosciences, Cat# 566121, RRID:AB_2739523
CD56	Brilliant Violet 605	NCAM16.2	BD Biosciences, Cat# 562780, RRID:AB_2728700
CD14	Brilliant Violet 786	M5E2	BD Biosciences, Cat# 563698, RRID:AB_2744287
CD4	FITC	SK3	BD Biosciences, Cat# 345768, RRID:AB_2868797
CD16	PE-Cy7	3G8	BD Biosciences, Cat# 557744, RRID:AB_396850
CD45	PerCP-Cy5.5	2D1	BD Biosciences, Cat# 332784, RRID:AB_2868632
CD22	APC	S-HCL-1	BD Biosciences, Cat# 333145, RRID:AB_2868646
CD3	APC H7	SK7	BD Biosciences, Cat# 641415, RRID:AB_2870309
CAT19 idiotype	PE	-	Autolus Therapeutics

APC, allophycocyanin; Cy, cyanine; FITC, fluorescein isothiocyanate; PE, R-phycoerythrin; PerCP, peridinin-chlorophyll-protein; RRID, Research Resource Identifiers.
