# Supplementary material for: CAR T-cell Kinetics, Persistence, and Clinical Outcomes in Adult Patients with Relapsed/Refractory B-cell ALL Treated with Obecabtagene Autoleucel in the FELIX Study
Source: Cancer Res Commun. 2026 Jul 15;6(7):1681–92. doi: 10.1158/2767-9764.CRC-25-0756 (PMC13370329; doi:10.1158/2767-9764.CRC-25-0756)
Supplement: Supplementary Table S2 — Intracellular flow cytometry surface antibody master mix [file crc-25-0756_supplementary_table_s2_suppst2.pdf]

Supplementary Table S2. Intracellular flow cytometry surface antibody master mix.

**Antigen**	**Fluorophore**	**Clone**	**Company, catalog and RRID**
Brilliant stain buffer plus	-	-	BD Biosciences, Cat# 566385, RRID:AB_2869761
CD45RO	Brilliant Violet 421	UCHL1	BD Biosciences, Cat# 562641, RRID:AB_2737696
CD45RA	Brilliant Violet 480	HI100	BD Biosciences, Cat# 566114, RRID:AB_2739516
CD8	Brilliant Violet 605	SK1	BD Biosciences, Cat# 564116, RRID:AB_2869551
CD27	Brilliant Violet 711	M-T271	BD Biosciences, Cat# 564893, RRID:AB_2739003
CD25	Brilliant Violet 786	M-A251	BD Biosciences, Cat# 563701, RRID:AB_2744338
CCR7	Alexa Fluor 488	G043H7	BioLegend, Cat# 353206, RRID:AB_10916389
PD-1	PE-Cy7	EH12.1	BD Biosciences, Cat# 561272, RRID:AB_10611585
CD4	PerCP-Cy5.5	SK3	BioLegend, Cat# 344608, RRID:AB_1953236
CD3	APC H7	SK7	BD Biosciences, Cat# 641415, RRID:AB_2870309

APC, allophycocyanin; Cy, cyanine; PE, R-phycoerythrin; PerCP, peridinin-chlorophyll-protein; RRID, Research Resource Identifiers.
