# Supplementary material for: CAR T-cell Kinetics, Persistence, and Clinical Outcomes in Adult Patients with Relapsed/Refractory B-cell ALL Treated with Obecabtagene Autoleucel in the FELIX Study
Source: Cancer Res Commun. 2026 Jul 15;6(7):1681–92. doi: 10.1158/2767-9764.CRC-25-0756 (PMC13370329; doi:10.1158/2767-9764.CRC-25-0756)
Supplement: Supplementary Table S3 — Intracellular flow cytometry master mix [file crc-25-0756_supplementary_table_s3_suppst3.pdf]

Supplementary Table S3. Intracellular flow cytometry master mix.

Antigen	Fluorophore	Clone	Company, catalog and RRID
1X Permeabilization solution	-	-	Thermo Fisher Scientific, Cat#00-5523-00
CAT19	PE	-	Autolus Therapeutics
FOXP3	APC/Alexa Fluor 647	PCH101	Thermo Fisher Scientific, Cat# 17-4776-42, RRID:AB_1603280
		259D	BioLegend, Cat# 320214, RRID:AB_492984
		150D	BioLegend, Cat# 320214, RRID:AB_492984

APC, allophycocyanin; PE, R-phycoerythrin; RRID, Research Resource Identifiers.
