# Supplementary material for: CAR T-cell Kinetics, Persistence, and Clinical Outcomes in Adult Patients with Relapsed/Refractory B-cell ALL Treated with Obecabtagene Autoleucel in the FELIX Study
Source: Cancer Res Commun. 2026 Jul 15;6(7):1681–92. doi: 10.1158/2767-9764.CRC-25-0756 (PMC13370329; doi:10.1158/2767-9764.CRC-25-0756)
Supplement: Supplementary Table S4 — Fluorescence minus multiple controls for setting gating limits [file crc-25-0756_supplementary_table_s4_suppst4.pdf]

Supplementary Table S4. Fluorescence minus multiple controls for setting gating limits.

Fluorescence minus control	Target antibodies
FMX1	CD27, CD45RA
FMX2	CCR7, CD25
FMX3	FOXP3, CAR
FMX4	PD1, CD45RO
