# Supplementary material for: CAR T-cell Kinetics, Persistence, and Clinical Outcomes in Adult Patients with Relapsed/Refractory B-cell ALL Treated with Obecabtagene Autoleucel in the FELIX Study
Source: Cancer Res Commun. 2026 Jul 15;6(7):1681–92. doi: 10.1158/2767-9764.CRC-25-0756 (PMC13370329; doi:10.1158/2767-9764.CRC-25-0756)
Supplement: Supplementary Table S5 — Summary of L-Psi duplex ddPCR assay [file crc-25-0756_supplementary_table_s5_suppst5.pdf]

Supplementary Table S5. Summary of L-Psi duplex ddPCR assay.

**Primer and probe sequences**	**Sequence, 5′–3′**
L-Psi forward primer	CTCTCTCGACGCAGGACTC
L-Psi reverse primer	TTTGGCGTACTCACCAGTCG
L-Psi probe	CTCTTGCCGTGCGCGCTTCAGCAAGC
RPP30 forward primer	AGTAACTTGTAAGTGGTAGTGCATAGA
RPP30 reverse primer	ATGTCAAGAGTAGGAGGACATTTG
RPP30 probe	TCAGGCAGACTGACACTAGAGTTC
**Parameter**	**L-Psi/RPP30 assay**
Optimal annealing temperature	59°C
Limit of detection*	11 copies†
Limit of quantification*	21 copies†
Linear range*	11–89,600 copies
Repeatability	1.6%–16.4%
Intermediate precision	1.6%–27.6%
ddPCR thresholds	FAM: L-Psi 1 assay: 1250<br>HEX: RPP30 assay: 1250
**Reaction Mix Components**	**Volume per reaction**
Supermix for probes (no dUTP)	10 µL
LPSI/RPP30_NA.001_Smix	2 µL
HindIII restriction enzyme	1 µL
gDNA	200 ng
Nuclease-free water	Up to 20 µL final volume
**Total**	20 µL

*L-Psi assay in duplex with RPP30 assay. †When providing merged-well data of two replicate measurements on the clinical samples and setting a threshold of five copies for a blank sample (maximum tolerated copies in a negative sample).

ddPCR, droplet digital PCR; dUTP, deoxyuridine triphosphate; FAM, fluorescein; gDNA, genomic DNA; HEX, hexachlorofluorescein; L-Psi, lentiviral-Psi.
