# Supplementary material for: CAR T-cell Kinetics, Persistence, and Clinical Outcomes in Adult Patients with Relapsed/Refractory B-cell ALL Treated with Obecabtagene Autoleucel in the FELIX Study
Source: Cancer Res Commun. 2026 Jul 15;6(7):1681–92. doi: 10.1158/2767-9764.CRC-25-0756 (PMC13370329; doi:10.1158/2767-9764.CRC-25-0756)
Supplement: Supplementary Table S6 — Summary of CAR T-cell persistence and BCA status at Month 3 and 6 [file crc-25-0756_supplementary_table_s6_suppst6.pdf]

Supplementary Table S6. Summary of CAR T-cell persistence and BCA status at Month 3 and 6.

Status, n (%)*	Month 3			Month 6		
	B-cell recovery	Ongoing BCA	Total	B-cell recovery	Ongoing BCA	Total
**Loss of persistence**	7 (36.8)	12 (63.2)	19	14 (77.8)	4 (22.2)	18
**Ongoing persistence**	2 (3.3)	58 (96.7)	60	0 (0)	42 (100)	42
**Total**	9	70	79	14	46	60

*Percentages were calculated using the total numbers of patients with loss of CAR T-cell persistence or ongoing persistence at Month 3 and Month 6.

BCA, B-cell aplasia; CAR, chimeric antigen receptor.
