# Supplementary material for: CAR T-cell Kinetics, Persistence, and Clinical Outcomes in Adult Patients with Relapsed/Refractory B-cell ALL Treated with Obecabtagene Autoleucel in the FELIX Study
Source: Cancer Res Commun. 2026 Jul 15;6(7):1681–92. doi: 10.1158/2767-9764.CRC-25-0756 (PMC13370329; doi:10.1158/2767-9764.CRC-25-0756)
Supplement: Supplementary Figure S1 — Kaplan-Meier landmark analysis of EFS among patients with ongoing remission without new anti-cancer therapies, by CAR T-cell persistence by intracellular flow cytometry at A) Month 3 and B) Month 6. [file crc-25-0756_supplementary_figure_s1_suppsf1.pdf]

**A**

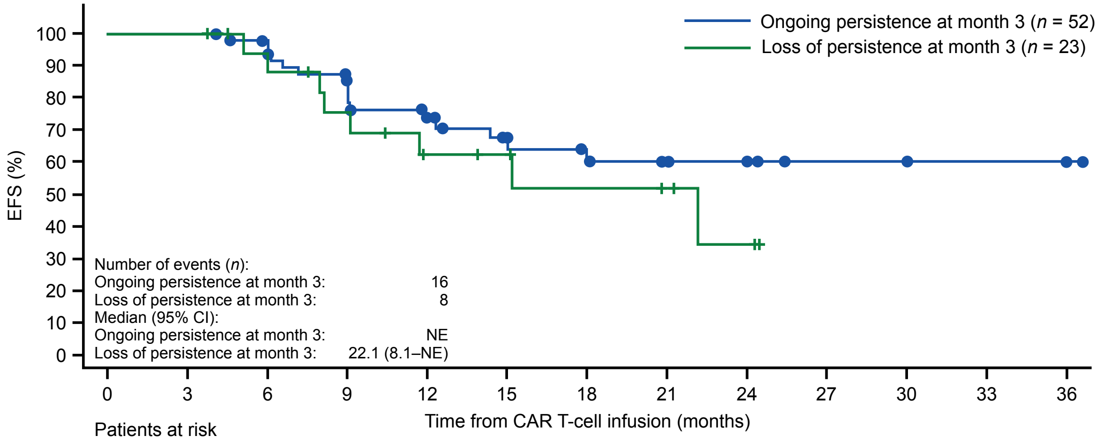


| Patients at risk | | | | | | | | | | | | | |
|---|---|---|---|---|---|---|---|---|---|---|---|---|---|
| Ongoing persistence at month 3 | 52 | 52 | 46 | 38 | 27 | 18 | 16 | 9 | 6 | 3 | 2 | 2 | 1 |
| Loss of persistence at month 3 | 23 | 23 | 16 | 12 | 8 | 7 | 5 | 4 | 2 | 0 | 0 | 0 | 0 |

**B**

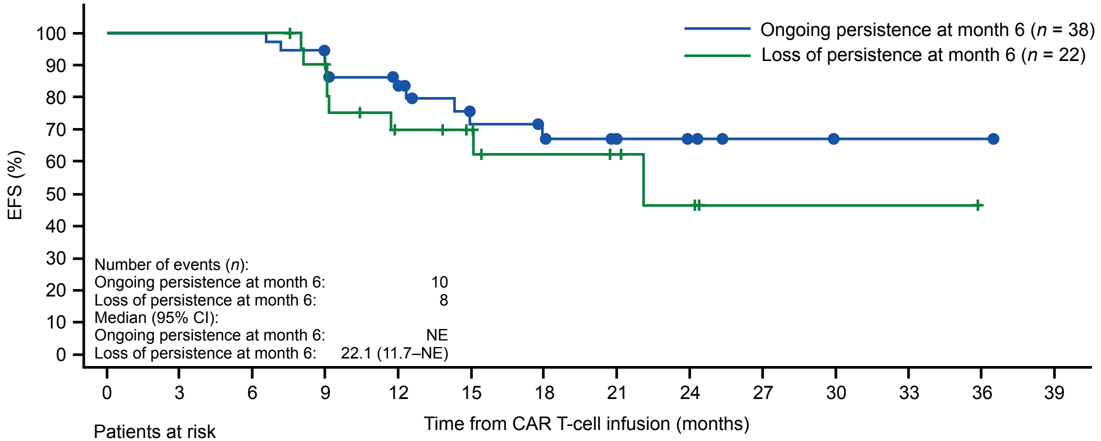


| Patients at risk | | | | | | | | | | | | | | |
|---|---|---|---|---|---|---|---|---|---|---|---|---|---|---|
| Ongoing persistence at month 6 | 38 | 38 | 38 | 34 | 25 | 17 | 15 | 8 | 5 | 2 | 1 | 1 | 1 | 0 |
| Loss of persistence at month 6 | 22 | 22 | 22 | 18 | 12 | 10 | 7 | 6 | 3 | 1 | 1 | 1 | 0 | 0 |

**Supplementary Figure 1.** Kaplan-Meier landmark analysis of EFS among patients with ongoing remission without new anticancer therapies, by CAR T-cell persistence by intracellular flow cytometry at (**A**) month 3 and (**B**) month 6.
CAR, chimeric antigen receptor; CI, confidence interval; EFS, event-free survival; NE, not estimable.
